# Single- and two-phase flow simulation based on equivalent pore network extracted from micro-CT images of sandstone core

**DOI:** 10.1186/s40064-016-2424-x

**Published:** 2016-06-21

**Authors:** Rui Song, Jianjun Liu, Mengmeng Cui

**Affiliations:** School of Geoscience and Technology, Southwest Petroleum University, Chengdu, 610500 China; State Key Laboratory of Oil and Gas Reservoir Geology and Exploitation, Southwest Petroleum University, Chengdu, 610500 China; School of Petroleum and Natural Gas Engineering, Southwest Petroleum University, Chengdu, 610500 China

**Keywords:** Image digitization based algorithm, Pore network, Micro-CT image, Lattice-Boltzmann method, Two-phase flow

## Abstract

Due to the intricate structure of porous rocks, relationships between porosity or saturation and petrophysical transport properties classically used for reservoir evaluation and recovery strategies are either very complex or nonexistent. Thus, the pore network model extracted from the natural porous media is emphasized as a breakthrough to predict the fluid transport properties in the complex micro pore structure. This paper presents a modified method of extracting the equivalent pore network model from the three-dimensional micro computed tomography images based on the maximum ball algorithm. The partition of pore and throat are improved to avoid tremendous memory usage when extracting the equivalent pore network model. The porosity calculated by the extracted pore network model agrees well with the original sandstone sample. Instead of the Poiseuille’s law used in the original work, the Lattice-Boltzmann method is employed to simulate the single- and two- phase flow in the extracted pore network. Good agreements are acquired on relative permeability saturation curves of the simulation against the experiment results.

## Background

Accurate acquisition of the micro structure and flow properties of porous media is of great importance in petroleum engineering, biomedical science, micro-electronics, and composites (Hilfer and Zauner 2011; Sanya et al. 2014; Tagar et al. 2016). Thus, the pore-scale modeling, where fluid displacement is simulated in a model of porous medium, has been used as a platform to study multi-phase flow at the pore scale.

The pore network was described by Fatt (1956a, b, c) for the first time. He randomly assigned radii to the two-dimensional (2D) regular lattice and predicted the capillary pressure and relative permeability of drainage. Since then, extensive studies on pore network models have been carried out to develop the predictive models of multi-phase flow focusing on the topology of the structure, pore size distribution, throats and their spatial correlation (Chatzis and Dullien 1977; Jerauld and Salter 1990; Lowry and Miller 1995). However, these realistic pore networks are consequently unable to reflect the real topology and complex geometry of natural porous media. In most of these studies, flow governing equations are obtained from the Poiseuille’s law. To accurately describe the flow conductance, the Poiseuille radius and length should correctly reflect the real pore and throat shape (Oren et al. 1998; Sholokhova et al. 2009). With the development of imaging technology, the three-dimensional (3D) images with resolution at the micron scale can be acquired by various approaches, such as micro computed tomography scanning technique (micro-CT), scanning electron microscopy (SEM), serial sectioning, confocal laser scanning microscopy, and reconstructed porous media by mathematical methods. Benefited from this, the reconstruction of pore network representing real rock structures has been extended from 2D thin sections (Laroche and Vizika 2005; Liu and Song 2015) or numerical reconstructions (Blunt 1998, 2001) to three-dimensional (3D) images at the micron scale (Bauer et al. 2012; Liu et al. 2015) currently.

Based on the maximum ball algorithm, a modified method of extracting the equivalent pore network model from the three-dimensional micro computed tomography images is presented in this paper. The equivalent pore network model is able to reproduce the intricate micro structure of the natural porous media sample properly. Based on the equivalent pore network model, simulation codes on single- and two-phase flow using the Lattice-Boltzmann method are developed to predict the flow properties. Meanwhile, the effects of wettability on oil recovery are studied.

## Pore network model extraction algorithm

A improved method of extracting the equivalent pore network model from the three-dimensional micro computed tomography (micro-CT) images based on the maximum ball algorithm (Silin and Patzek 2006) is proposed in this paper. In the original work, the maximal ball algorithm starts from each voxel in the pore space to find the largest inscribed spheres that just touch the grain or the boundary, and the pore and throat are segmented by ranking the local radii of the spheres (Silin and Patzek 2006), which consume tremendous memory to find the maximum spheres and remove the smaller balls included by larger ones. Here, some improvements are made to simplify the process of building the maximum spheres and the partition of pores and throats. The workflow of this algorithm can be summarized in the following steps.

1. Image acquire and segmentation. In this paper, two sandstone samples named by ST1 and ST2 from Shengli Oilfield in China are imaged in the State Key Laboratory of Oil and Gas Reservoir Geology and Exploitation in Southwest Petroleum University and used an input to the equivalent pore network model reconstruction. Image segmentation is a crucial step in image analysis to turn the grey scales of the original micro-CT image into black and white representing the void and solid. Meanwhile, the median filter algorithm is used to remove noise, especially the salt and pepper noise which causes tiny and isolate pores in the model. The 3D segmented and filtered images of ST1 and ST2 containing 400^3^ pixels are shown in Fig. 1, in which the black part represents the pore and the cyan part is the matrix.

**Fig. 1:**
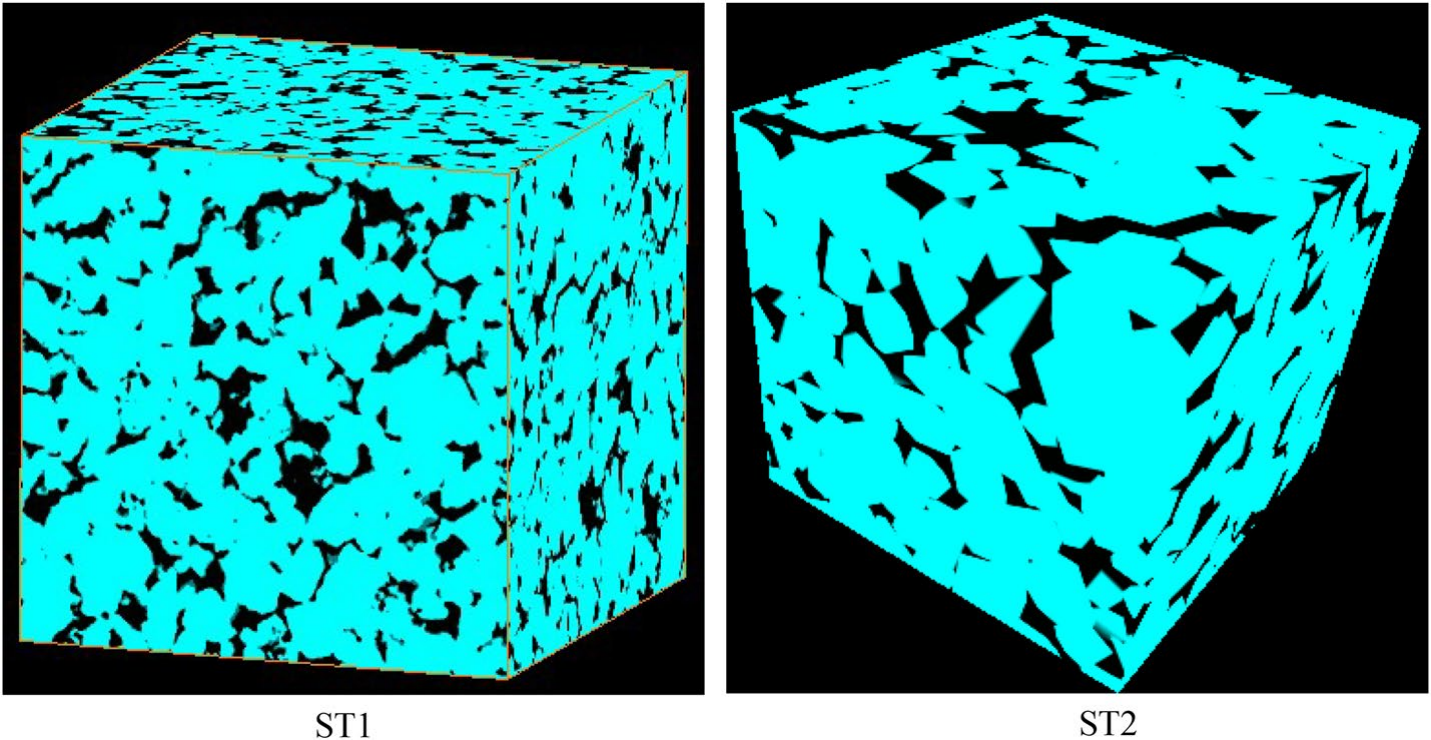
3D segmented image of ST1 and ST2. The *black part* is the pore and the *cyan part* is the matrix
2. Medial axis extraction and the pore and throat partition. The medial axis can be obtained by a pore space burning algorithm (Lindquist et al. 1996; Lindquist and Venkatarangan 1999), and the burn number is recorded for each medial axis, which defines the radius of maximum spheres. Then the maximum spheres are centered along the medial axis. The intersection of greater than or equal to three median axes and the balls on the boundary are defined as pores, and the others are throats. The throats are replaced by cylinders with the average radius of the biggest balls in the pore-throat chains linking two pores. The minimum size of a pore is defined in codes. For example, a pore space containing two or less voxels is defined as a throat, but cannot be pore in numerical simulation in this paper. The median axis and initial partition of ST1 is illustrated in Fig. 2, in which the line represents the throat and the small ball represents the pore. The extracted equivalent pore network model of ST1 and ST2 are illustrated in Fig. 3.

**Fig. 2:**
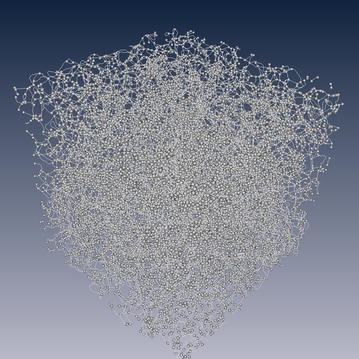
Median axis and initial partition of pores and throats. The *lines* represents the throat and the *small balls* represent pores

**Fig. 3:**
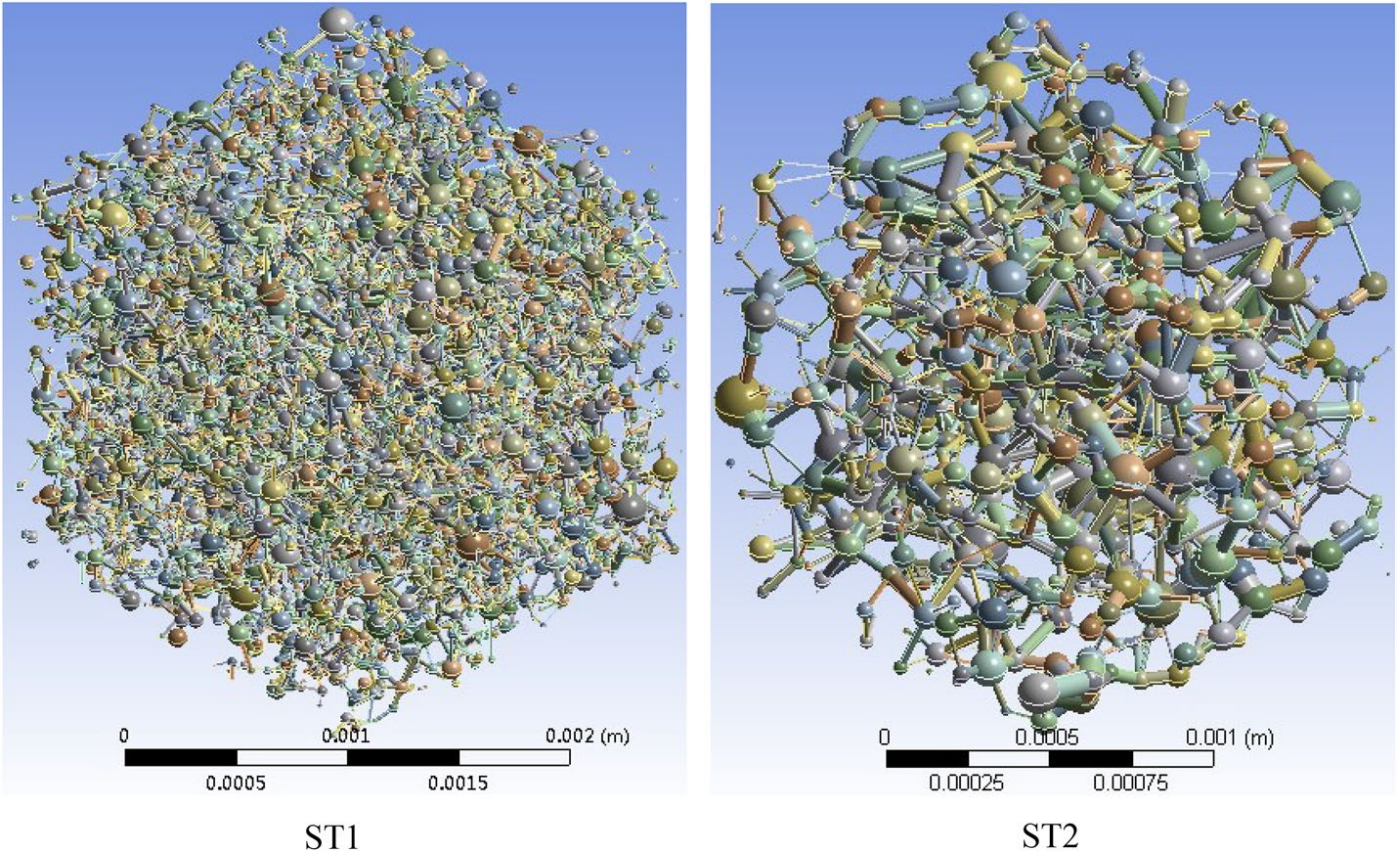
EPNMs of ST1 and ST2. *Different color* in the *image* represents different radii

## LBGK mathematical model

In recent years, the Lattice-Boltzmann method (LBM) has been developed into an alternative and promising numerical scheme for the simulation of fluid flows and physics modeling in fluids. The scheme is particularly effective in fluid flow applications involving interfacial dynamics and complex boundaries (porous media, e.g.) (Chen and Doolen 1998). Several algorithms have been developed since the Lattice-Boltzmann method was proposed by Lugwig Boltzmann in 1872, among which, the Bhatnagar–Gross–Krook (BGK) collision term (Qian 1993; Chen et al. 1995) has been treated as a popular algorithm in field of shock formation, multi-phase flow, and porous material property test (Crouse et al. 2002; Luan et al. 2011; Gooneratne et al. 2013). The Lattice Bhatnagar–Gross–Krook (LBGK) model used in this paper can be expressed as:

1 $$f_{i} \left( {\overrightarrow {x} + \overrightarrow {c}_{i} ,t + 1} \right) - f_{i} \left( {\overrightarrow {x} ,t} \right) = \varOmega_{i}^{j} (x,t)$$

where $f_{i} (\overrightarrow {x} ,t)$ is the particle distribution function at location *x* and time *t* along the *i*th direction (*i* = 0, 1, 2…18) using 3D nineteen velocity model; $\varOmega_{i}^{j} (x,t)$ is the collision term; $\overrightarrow {c}_{i}$ is the velocity vector.

The two phase flow mathematical model is the color-gradient model proposed by Gunstensen et al. (1991). For a two-phase flow, the local fluid particle distribution *f*_*i*_ is defined as two parts (Gunstensen et al. 1991),

2 $$\begin{aligned} f_{j} & = f_{j}^{R} + f_{j}^{B} \\ f_{j}^{R} & = \frac{{\rho_{R} }}{{\rho_{R} + \rho_{B} }}f_{j} + \beta \frac{{\rho_{R} \rho_{B} }}{{(\rho_{R} + \rho_{B} )^{2} }}f_{i}^{(eq,0)} \cos \nu_{f} \\ f_{j}^{B} & = \frac{{\rho_{B} }}{{\rho_{R} + \rho_{B} }}f_{j} - \beta \frac{{\rho_{R} \rho_{B} }}{{(\rho_{R} + \rho_{B} )^{2} }}f_{i}^{(eq,0)} \cos \nu_{f} \\ \end{aligned}$$

where $f_{i}^{R}$ and $f_{i}^{B}$ refer to the red and blue fluid in the two-phase system; *ρ*_*R*_ and *ρ*_*B*_ are the fluid density of red and blue color; *ν* is the angle of local color gradient versus the lattice calculated direction; $f_{i}^{eq}$ is a quadratic expansion of the Maxwell–Boltzmann distribution; *β* represents the separation trends of the two immiscible fluids between 0 and 1.

By introducing the surface tension term, the collision term can be calculated by (Gunstensen et al. 1991),

3 $$\varOmega_{i}^{j} = - \frac{1}{\tau }\left( {f_{i} - f_{i}^{eq} } \right) + \sigma \cos 2(\nu_{i} - \nu_{f} )\sum\limits_{i} {\overset{\lower0.5em\hbox{$\smash{\scriptscriptstyle\rightharpoonup}$}}{{c_{i} }} [\rho_{R} (x + \overset{\lower0.5em\hbox{$\smash{\scriptscriptstyle\rightharpoonup}$}}{{c_{i} }} ) - \rho_{B} (x + \overset{\lower0.5em\hbox{$\smash{\scriptscriptstyle\rightharpoonup}$}}{{c_{i} }} )]}$$

where *τ* is the single time relaxation parameter; *σ* is the surface tension; *ν*_*i*_ is the angle of the *i*th direction of the lattice.

The conserved quantities of mass and momentum are calculated by,

4 $$\rho = \sum\limits_{i} {f_{i}^{0} }$$

5 $$\rho \overrightarrow {u} = \sum\limits_{i} {f_{i}^{0} } \overrightarrow {e}$$

where *ρ* and *u* is the density and the local velocity, respectively.

The interaction between different fluid phases is defined as (Ramstad et al. 2010),

6 $$F_{\text{int}} (x,t) = - G\psi (x,t)\sum\limits_{a} {\omega_{a} } \psi (x + \overrightarrow {{e_{i} }} \Delta t)\overrightarrow {{e_{i} }}$$

where *G* is a parameter that controls the strength of the inter-particle force.

When we assume the density of rock is *ρ*_*w*_, the wettability of the rock is defined by the interaction between the solid and fluids, and can be described as (Ramstad et al. 2010),

7 $$F_{\sigma } (x) = - G_{\sigma } \psi (\rho (x,t))\sum\limits_{i = 0} {\omega_{i} \psi (\rho_{w} )s(x + \overrightarrow {{e_{i} }} \Delta t)\overrightarrow {{e_{i} }} }$$

where *ω*_*i*_ is the weight coefficient; $s(x + \overrightarrow {{e_{i} }} \Delta t)$ is an indicator function that is equal to 1 or 0 for a solid or a fluid domain node, respectively; *ρ*_*w*_ is used to represents the different wall properties for different contact angles.

Then the macroscopic flow rate *Q*_*j*_ can be calculated to determine the intrinsic permeability. The intrinsic permeability *K* of the network is obtained from Darcy’s law at complete saturation. The absolute permeability *K* of the network is derived from Darcy’s law,

8 $$K = \frac{{\mu_{j} Q_{j} L}}{A\Delta P}$$

where the network is fully saturated with a single phase *j* of viscosity *μ*; *Q*_*j*_ is the total single phase flow rate through the pore network of length *L* with the potential drop *ΔP*; *A* is the cross-sectional area of the model.

Then relative permeability is

9 $$K_{r} = \frac{{Q_{jt} }}{{Q_{j} }}$$

where *Q*_*jt*_ is the total flow rate of phase *j* in multiphase conditions with the same imposed pressure drop *ΔP*.

## Results

Some basic parameters of the pore network models are listed in Table 1. It is shown that the porosity of the equivalent pore network models is close to experimental data of the original sandstone samples in the case of that the spheres volume is equal to the original pore space. Based on the extracted equivalent pore network models and LBM, the simulation codes for single- and two- phase flow are developed. As is listed in Table 2, the absolute permeability of the equivalent pore network models are computed and compared to the experimental data, in which good agreement is acquired between the absolute permeability of the equivalent pore network models and the experimental benchmark data.

**Table 1:** Basic parameters of the models

	Size (mm)	Number of pores	Number of throats	Average connection number	Porosity of EPNM (%)	Experimental porosity (%)
ST1	2.14	6298	12,558	3.92	19.27	18.73
ST2	1.46	810	1563	3.72	13.71	13.11

**Table 2:** Absolute permeability of EPNMs

	EPNM/mD	MB/mD
ST1	1210	1100
ST2	872	703

**Fig. 4:**
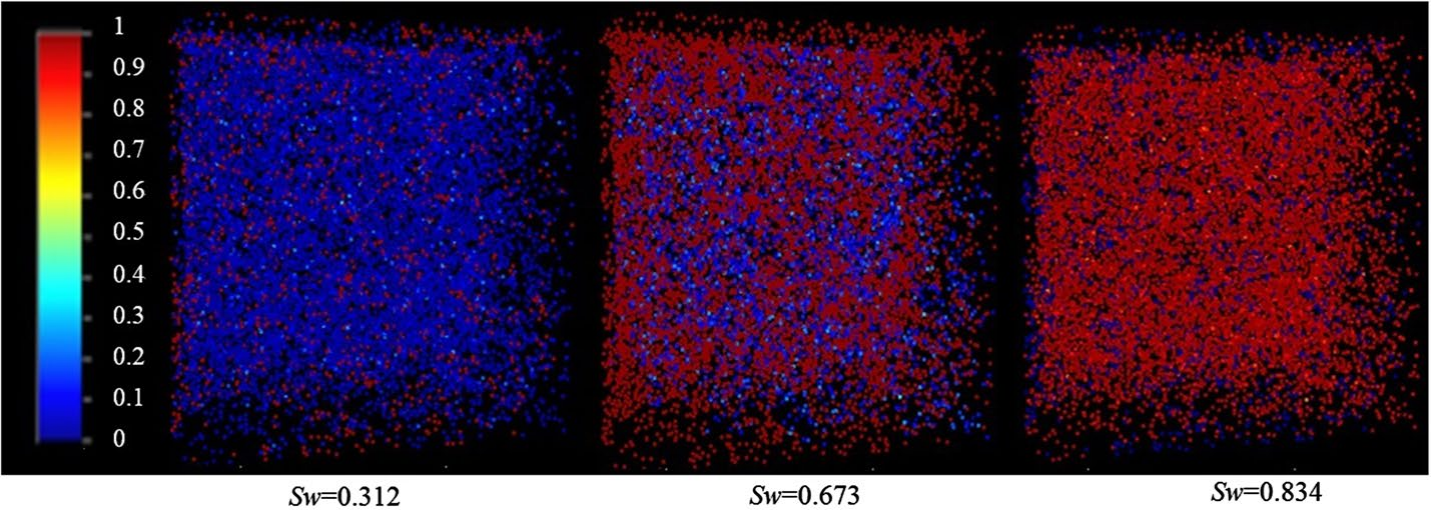
Water volume fraction distribution of ST1 for different water saturation. *Each point* represents the center of pore

In the water flooding process, the inlet or outlet boundaries have been selected the same as the actual experimental water flooding process, which means, the top and bottom of the model is regarded as the inlet and outlet respectively, while the other boundaries are treated as impermeable. The model is initially saturated by oil and water is injected from the inlet. Fluid parameters used in the simulation are listed in Table 3. Water volume fraction distribution for different saturation in the water flooding process of the extracted pore network is shown in Fig. 4, from which it is found that the oil is displaced in the pore space along with the water invading from the inlet gradually. Oil and water move along the respective channel to reduce the flow resistance, and it is rare for oil and water to flow alternate in the same pore. The displacement lag is remarkable. This phenomenon is called viscous fingering caused by fluid flowing along the lower flow resistance channels. Meanwhile, oil in tiny throats is impossible to be displaced by water because of the tremendous capillary force. To show the displacement process in detail, the water volume fraction distribution is illustrated in this paper.

**Table 3:** Fluid parameters used in the simulation

Interfacial tension *σ* (mN/m)	Water viscosity *μ*_*w*_ (cp)	Oil viscosity *μ*_*o*_ (cp)	Water density *ρ*_*w*_ (kg/m^3^)	Oil density *ρ*_*o*_ (kg/m^3^)	*p*_*inlet*_ (Pa)	*p*_*outlet*_ (Pa)
48.0	0.991	5.23	1000	890	10	0

**Fig. 5:**
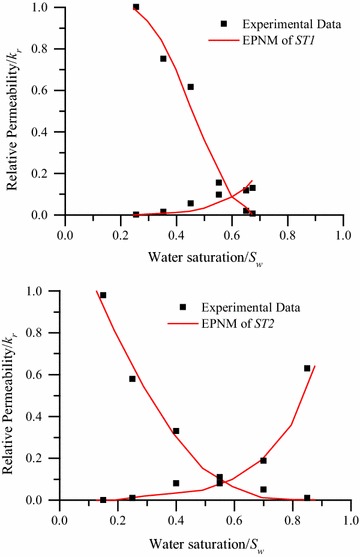
Permeability saturation curves of both the EPNMs and the experimental results

In the simulation, the intrinsic contact angle is assigned as a random distribution to pores and throats in the network in a distribution interval. By adopting the average distribution of intrinsic contact angle intervals, different wetting systems from water-wet to oil-wet can be obtained. Considering that the original sandstones are water-wettable, the contact angle used in the simulation is assigned as [30–60]°. Meanwhile, the permeability saturation curves of both the experiment and simulation results for different wettability are plotted in Fig. 5, which also verifies the feasibility of the equivalent pore network model and the two-phase code.

Wettability of the reservoir rock directly affects the flow of fluids, and fluids distribution in a reservoir, which is important for transport properties determination. Many experimental investigations on the impact of wettability have been conducted. However, it is impossible to obtain two natural rock cores with the same micro structure and the opposite wettability, that is, it is difficult to realize the single variable control of wettability at the laboratory scale. Simulation of water flooding in the extracted equivalent pore network model can be an effective method to solve this problem.

**Fig. 6:**
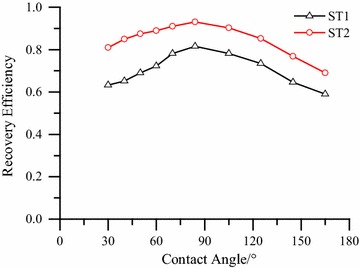
Recovery efficiency V.S. Average contact angle *θ*_*i*_

In this paper, different wettability conditions of the same pore network (ST1 and ST2) are assigned to study the effects on oil recovery. As is shown in Fig. 6, the optimal oil recovery is found in mixed wettability sandstone, when the contact angle is in the interval [80, 100]°. This trend of the simulation results is identical to the experimental result by Morrow et al. (1986) and Jadhunandan et al. (1995). Combined with the permeability saturation curves in Fig. 6, it is found that the water relative permeability and the residual oil saturation increase with the contact angle in [0, 90]° and decrease with the increase of the contact angle in [90, 180]°. In water-wet media, water can flow readily through the wetting layers in the corners of the pore space, while oil is trapped in the larger pores. Then the increase of contact angle leads to a connected oil flowing channel and less trapping. When the medium becomes oil-wet, oil attaches to the solid surface and viscous fingering occurs for water in the pore space, which will reduce the effective channel radius of water and oil recovery.

## Conclusion

This paper presents a modified method of extracting equivalent pore network model from the 3D micro-CT images based on the maximum ball algorithm. The improved partition methods are able to avoid tremendous memory usage when extracting the equivalent pore network model. Two types of micro sandstone images are used to display the pore network models and simulate for single- and two- phase flow. The porosity, calculated permeability of the pore network model agree well with experimental benchmark data of the original sandstone sample. Using the extracted pore network model and two-phase flow codes based on Lattice Boltzmann method, the simulation on water flooding mechanism is conducted to obtain the effects of wettability on oil recovery in the porous sandstone. Visualized water flooding process and the relative permeability saturation curves are obtained. Moreover, it is found that the optimal oil recovery would be realized in the mixed wettability reservoirs. Both of the two simulation results are identical to the experimental benchmark data, which verifies the feasibility of our pore network model and the simulation codes.

However, the assumptions used in the pore network extraction algorithm, which leads to idealized shape, radius and connectivity of pore and throat, result in a lager porosity and permeability of the reconstructed model compared to the original sample. A further research focus on the reproduction of the pore structure for the real shape is worth of studying to realize a wider application in other fields.

## Abbreviations

3D: three-dimensional
BGK: Bhatnagar–Gross–Krook
EPNM: equivalent pore network model
LBGK: Lattice Bhatnagar–Gross–Krook
LBM: Lattice Boltzmann method
Micro-CT: micro computed tomography
SEM: scanning electron microscopy

## References

[CR1] Bauer D, Youssef S, Fleury M, Bekri S, Rosenberg E, Vizika O (2012). Improving the estimations of petrophysical transport behavior of carbonate rocks using a dual pore network approach combined with computed micro tomography. Transp Porous Media.

[CR2] Blunt MJ (1998). Physically-based network modeling of multiphase flow in intermediate-wet porous media. J Petrol Sci Eng.

[CR3] Blunt MJ (2001). Flow in porous media pore-network models and multiphase flow. Curr Opin Colloid Interface Sci.

[CR4] Chatzis I, Dullien FAL (1977). Modeling pore structure by 2-D and 3-D networks with application to sandstones. J Can Pet Technol.

[CR5] Chen S, Doolen GD (1998). Lattice Boltzmann method for fluid flows. Annu Rev Fluid Mech.

[CR6] Chen Y, Ohashi H, Akiyama M (1995). Prandtl number of lattice Bhatnagar–Gross–Krook fluid. Phys Fluids.

[CR7] Crouse B, Krafczyk M, Kühner S, Rank E, van Treeck C (2002). Indoor air flow analysis based on lattice Boltzmann methods. Energy Build.

[CR8] Fatt I (1956). The network model of porous media I. Capillary pressure characteristics. Trans AIME.

[CR9] Fatt I (1956). The network model of porous media II. Dynamic properties of a single size tube network. Trans AIME.

[CR10] Fatt I (1956). The network model of porous media III. Dynamic properties of networks with tube radius distribution. Trans AIME.

[CR11] Gooneratne CP, Kurnicki A, Yamada S, Mukhopadhyay SC, Kosel J (2013). Analysis of the distribution of magnetic fluid inside tumors by a giant magnetoresistance probe. PLoS One.

[CR12] Gunstensen AK, Rothman DH, Zaleski S, Zanetti G (1991). Lattice Boltzmann model of immiscible fluids. Phys Rev A.

[CR13] Hilfer R, Zauner T (2011). High-precision synthetic computed tomography of reconstructed porous media. Phys Rev E.

[CR14] Jadhunandan PP, Morrow NR (1995). Effect of wettability on waterflood recovery for crude-oil/brine/rock systems. SPE Reserv Eng.

[CR15] Jerauld GR, Salter SJ (1990). The effects of pore-structure on hysteresis in relative permeability and capillary pressure: pore-level modeling. Transp Porous Media.

[CR16] Laroche C, Vizika O (2005). Two-phase flow properties prediction from small-scale data using pore-network modeling. Transp Porous Media.

[CR17] Lindquist WB, Venkatarangan A (1999). Investigating 3D geometry of porous media from high resolution images. Phys Chem Earth Part A.

[CR18] Lindquist LWB, Lee SM, Coker DA, Jones KW, Spanne P (1996). Medial axis analysis of void structure in three-dimensional tomographic images of porous media. J Geophys Res Solid Earth.

[CR19] Liu J, Song R (2015). Investigation of water and CO_2_ flooding using pore-scale reconstructed model based on micro-CT images of Berea sandstone core. Prog Comput Fluid Dyn Int J.

[CR20] Liu JJ, Rui SONG, Cui MM (2015). Improvement of predictions of petrophysical transport behavior using three-dimensional finite volume element model with micro-CT images. J Hydrodyn Ser B.

[CR21] Lowry MI, Miller CT (1995). Pore-scale modeling of nonwetting-phase residual in porous media. Water Resour Res.

[CR22] Luan HB, Xu H, Chen L, Sun DL, He YL, Tao WQ (2011). Evaluation of the coupling scheme of FVM and LBM for fluid flows around complex geometries. Int J Heat Mass Transf.

[CR23] Morrow N, Lim H, Ward J (1986). Effect of crude-oil-induced wettability changes on oil recovery. SPE Form Eval.

[CR24] Oren PE, Bakke S, Arntzen OJ (1998). Extending predictive capabilities to network models. SPE J.

[CR25] Qian YH (1993). Simulating thermohydrodynamics with lattice BGK models. J Sci Comput.

[CR26] Ramstad T, Øren PE, Bakke S (2010). Simulation of two-phase flow in reservoir rocks using a lattice Boltzmann method. SPE J.

[CR27] Sanya AS, Akowanou C, Sanya EA, Degan G (2014). Liquid film condensation along a vertical surface in a thin porous medium with large anisotropic permeability. SpringerPlus.

[CR28] Sholokhova Y, Kim D, Lindquist WB (2009). Network flow modeling via lattice-Boltzmann based channel conductance. Adv Water Resour.

[CR29] Silin D, Patzek T (2006). Pore space morphology analysis using maximal inscribed spheres. Phys A.

[CR30] Tagar AA, Changying J, Qishuo D, Adamowski J, Malard J, Eltoum F (2016). Implications of variability in soil structures and physio-mechanical properties of soil after different failure patterns. Geoderma.

